# Mothers' knowledge and home care practices for children with autism spectrum disorder in Diwaniyah, Iraq

**DOI:** 10.3389/frcha.2026.1705556

**Published:** 2026-03-12

**Authors:** Aqeel Abd Al-Hamza Marhoon, Khamees Bandar Obaid

**Affiliations:** Department of Pediatric Nursing, College of Nursing, University of Babylon, Babylon, Iraq

**Keywords:** autism spectrum disorder, children, home care practices, Iraq, knowledge, mother

## Abstract

**Background:**

Autism spectrum disorder (ASD) poses significant caregiving challenges, particularly in low-resource and culturally diverse settings. This study evaluated maternal knowledge and home care practices related to children with ASD in Diwaniyah, Iraq*.*

**Methods:**

A cross-sectional census survey was conducted in 2025 among 205 mothers of children diagnosed with ASD who were registered at autism centers in Diwaniyah. Data were collected via a validated, interviewer-administered questionnaire that assessed maternal knowledge of ASD and home care practices across nutrition, hygiene, motor skills, and safety domains. Statistical analyses were performed using SPSS version 25, employing descriptive statistics and chi-square tests. A significance level of *p* < 0.05 was adopted for all inferential tests.

**Results:**

Overall maternal knowledge of ASD was moderate. Higher knowledge was significantly associated with maternal education (*p* < 0.001), urban residence (*p* = 0.009), family size (*p* = 0.029), monthly income (*p* = 0.041), and consanguinity (*p* = 0.017). Home care practices were generally adequate in nutrition, personal hygiene, and motor skill development; however, safety practices were markedly insufficient. Awareness of ASD-related safety risks was also low. A significant positive association was observed between maternal knowledge and home care practices across all domains (*p* < 0.001).

**Conclusion:**

Mothers in Diwaniyah demonstrate moderate knowledge and active caregiving in most domains, yet there is insufficient focus on child safety at home. These findings highlight the urgent need for culturally tailored educational programs that emphasize home safety, expand access to autism services, and integrate genetic counseling to improve care for children with ASD in resource-limited settings.

## Introduction

Autism spectrum disorder (ASD) is a lifelong neurodevelopmental condition characterized by persistent deficits in social communication and interaction, along with restricted and repetitive behaviors and interests (1). The global prevalence of ASD has risen notably in recent years, with the World Health Organization estimating that approximately 1 in every 100 children is affected (2). Although global awareness of ASD is increasing, significant disparities in early diagnosis, intervention services, and caregiver support remain, particularly in low- and middle-income countries (3). In Iraq, these disparities are compounded by decades of conflict, political instability, under-resourced healthcare infrastructure, and widespread societal stigma surrounding neurodevelopmental disorders (4). Specialized services for children with ASD are sparse and often concentrated in major urban centers, making access difficult for families living outside these areas. Even in urban regions like Diwaniyah, where this study was conducted, services remain fragmented, with few autism-specific educational programs or trained professionals available to support affected families. As a result, caregiving responsibilities fall heavily on parents, particularly mothers, who often manage their child's daily needs without adequate professional guidance or support.

In this context, home-based care becomes the most immediate and consistent form of intervention for children with ASD. Mothers are typically the primary caregivers and play a critical role in implementing daily routines that support their child's development in key areas such as nutrition, hygiene, motor skill acquisition, and safety. These caregiving practices are heavily influenced by the mother's level of knowledge about autism, its symptoms, and evidence-based management strategies. However, in Iraq, maternal knowledge about ASD remains variable and largely undocumented, especially at the regional level. Diwaniyah, located in southern Iraq, is a province with a growing population and a relatively high prevalence of consanguineous marriage, a factor linked to increased genetic risk for neurodevelopmental disorders (5, 6). Despite this, local healthcare and educational systems provide limited autism-specific services or awareness campaigns, and caregivers often rely on informal sources of information. Understanding the context of caregiving in Diwaniyah is essential for identifying the specific challenges faced by mothers and the factors that may support or hinder effective home care.

Existing research from other countries has highlighted the importance of maternal knowledge in improving caregiving outcomes for children with ASD (7, 8). For instance, Wang et al. (7) demonstrated a strong association between maternal knowledge and improved home care practices, while Othman et al. (8) found that caregiver training programs significantly enhanced parental skills in managing ASD-related behaviors. Conversely, studies in the Arab region have revealed persistent misconceptions about autism, with many parents attributing symptoms to supernatural or religious causes (9, 10). In Iraq, available research has primarily focused on diagnostic patterns or general public awareness of ASD (11), with limited attention paid to the specific knowledge and daily caregiving practices of mothers. Additionally, factors such as maternal education level, economic status, urban vs. rural residency, consanguinity, and family psychiatric history may influence knowledge levels and caregiving behaviors but remain underexplored in the Iraqi context.

Given the central role of mothers in the home care of children with ASD and the absence of localized data, there is a critical need to assess both their understanding of autism and their day-to-day caregiving practices. This is particularly important in Diwaniyah, where social, cultural, and economic factors may uniquely shape the caregiving environment. Therefore, this study aimed to assess mothers' knowledge and home care practices in four essential domains, namely, nutrition, personal hygiene, motor skills, and safety measures, for children diagnosed with ASD in Diwaniyah, Iraq. This study also sought to examine the associations between maternal knowledge, caregiving behaviors, and various sociodemographic and clinical characteristics. The findings will provide valuable insights for designing targeted educational programs and interventions aimed at improving caregiving quality and child outcomes in similar low-resource settings.

## Materials and methods

### Study design

This descriptive, cross-sectional study was conducted over 5 months from April to August 2025 to evaluate mothers' knowledge and home care practices related to nutrition, personal hygiene, motor skills, and safety measures for children diagnosed with ASD. The cross-sectional design was selected to provide a comprehensive snapshot of maternal knowledge and caregiving behaviors at the time of the study.

### Study setting

The study took place in Diwaniyah governorate, located in central-southern Iraq. Data were collected from the following three primary centers specializing in autism care and education: the Raja Governmental Institute, the Ruqaya Private Institute for ASD, and the Al-Sabatin Foundation for Autism and Growth Forests, affiliated with the Health and Medical Education Authority. These centers serve as major referral and treatment hubs for children with ASD from both urban and rural communities, offering diagnostic, therapeutic, and educational services tailored to this population.

### Inclusion and exclusion criteria

The participants included mothers of children aged 2–16 years with an official diagnosis of ASD who were registered at one of the three autism centers in Diwaniyah. Eligible mothers had to be willing to participate voluntarily and provide written informed consent. Mothers of children with severe intellectual disabilities or physical impairments that could significantly interfere with routine home care were excluded. Additionally, incomplete questionnaires or a lack of consent resulted in exclusion from the final analysis.

### Sample size and sampling strategy

A census sampling approach was employed, whereby all mothers who met the inclusion criteria and were registered at the participating centers during the study period were invited to participate. A total of 205 eligible mothers were identified and included, ensuring a comprehensive representation of the accessible population within these specialized centers, which strengthens the generalizability of the findings within the local context.

### Data collection

Data were gathered using a structured, interviewer-administered questionnaire divided into three sections. The first section collected sociodemographic and clinical information, including maternal age, education, residence, family size, income level, consanguinity status, and history of participation in ASD-related educational programs. The second section assessed maternal knowledge of ASD, focusing on an understanding of the disorder's definition, causes, symptoms, and management strategies. The third section explored home care practices across four domains, namely, nutrition, personal hygiene, motor skills, and safety measures. The items in the second and third sections were derived from established frameworks and prior validated studies in the field; in addition, permission was obtained to use the data collection tools (12, 13). The scoring system for maternal knowledge and home care practices was clearly defined, with total scores converted to percentages and categorized as low (<50%), moderate (50%–75%), or high/good (>75%) to reflect meaningful differences in understanding and engagement. This standardized approach, based on prior validated studies (12, 13), ensures transparent interpretation and allows consistent comparison across the different domains of care. The overall knowledge score was calculated by summing all individual knowledge items, with correct answers scored 1 and incorrect or “don't know” answers scored 0, and then converted to a percentage and categorized as low (<50%), moderate (50%–75%), or high (>75%). The overall home care practice score was obtained by summing the frequency-based responses across all items (never = 0, sometimes = 1, always = 2), converting to a percentage, and classifying as low (1–1.66), moderate (1.67–2.33), or high (>2.33), providing a standardized measure of maternal engagement in caregiving practices.

The questionnaire was adapted and culturally tailored through expert review by a panel of six specialists in pediatric nursing, child psychiatry, and autism therapy. It then underwent rigorous forward and backward translation to ensure linguistic and conceptual accuracy in Arabic; the forward translation was conducted by a bilingual healthcare professional, while the backward translation was performed independently by a second bilingual expert, with discrepancies resolved by consensus. Face-to-face interviews were conducted by trained researchers during routine visits to the centers, in private settings to maintain confidentiality and encourage honest responses. The study's purpose and procedures were clearly explained to the participants.

### Pilot study

A pilot test involving 20 mothers was conducted to evaluate the clarity, cultural relevance, and practicality of the questionnaire. Feedback from these participants led to minor adjustments in phrasing and format to improve comprehension. The reliability of the instrument was confirmed by Cronbach's alpha values of 0.87 for the maternal knowledge section and 0.83 for the home care practices section, indicating strong internal consistency. Data from the pilot phase were excluded from the main study analysis.

### Statistical analysis

Data were analyzed using SPSS version 25. Descriptive statistics, including frequencies, percentages, means, and standard deviations, were computed to summarize participant characteristics and questionnaire responses. The chi-square test was used to examine associations between maternal knowledge levels, caregiving practices, and sociodemographic or clinical variables. A significance level of *p* < 0.05 was adopted for all inferential tests.

### Ethical considerations

Ethical approval was granted by the Scientific Research Ethics Committee at the University of Babylon (Research Ethics Committee Issue No: 83; 21 April 2025). Written informed consent was obtained from all participating mothers after a thorough explanation of the study's aims, procedures, confidentiality, and voluntary nature. The participants were assured of their right to withdraw at any time without penalty. All data were handled confidentially and stored securely in compliance with ethical standards. The study was conducted with respect for cultural sensitivities and the emotional wellbeing of participants.

## Results

Table 1 outlines the sociodemographic and clinical profiles of the 205 participants involved in the study. Nearly half of the mothers (48.8%) were aged between 29 and 38 years, followed by 39.5% who were 39 years or older. A majority (80.4%) had completed at least a secondary level of education, with 26.7% holding a bachelor's degree and 4.4% having postgraduate qualifications. The majority of the participants (91.2%) lived in urban areas, and 58.0% reported having a family size of 3–5 members. Regarding economic conditions, 38.6% of families reported sufficient income, while 27.3% indicated it was insufficient. A psychiatric history was noted in 13.7% of families, and consanguineous marriages were reported by 38.0%.

**Table 1:** Sociodemographic and clinical characteristics of the study participants (*n* = 205).

Variable	Category	Frequency (*n*)	Percentage (%)
Mother's age (years)	18–28	24	11.7
	29–38	100	48.8
	≥39	81	39.5
Education level	Cannot read/write	8	3.9
	Reads and writes	20	9.8
	Elementary	12	5.9
	Secondary	58	28.3
	Diploma	43	21.0
	Bachelor	55	26.7
	Postgraduate	9	4.4
Place of residence	Urban	187	91.2
	Rural	18	8.8
Family size	3–5 members	119	58.0
	6–9 members	50	24.4
	≥10 members	36	17.6
Monthly income	Not enough	56	27.3
	Somewhat enough	70	34.1
	Enough	79	38.6
Family psychiatric history	Yes	28	13.7
	No	177	86.3
Consanguineous marriage	Yes	78	38.0
	No	127	62.0
Attended autism education sessions	Yes	29	14.1
	No	176	85.9
Child's gender	Male	133	64.9
	Female	72	35.1
Child's age (years)	2–7	129	62.9
	8–13	61	29.8
	14–16	15	7.3
Birth order of the child with autism	Only child	8	3.9
	First	78	38.0
	Second	26	12.7
	Third or more	93	45.4
Severity of autism	Mild	52	25.4
	Moderate	124	60.5
	Severe	29	14.1
Duration since diagnosis	1–3 years	66	32.2
	4–6 years	68	33.2
	≥7 years	71	34.6

The majority of the mothers (85.9%) had not attended any educational sessions related to ASD. In terms of clinical characteristics, 64.9% of the children with ASD were male and the majority of the children (62.9%) were aged 2–7 years. Furthermore, approximately 45.4% were third-born or later, while 3.9% were the only child. The severity of ASD was classified as moderate in 60.5% of cases, mild in 25.4%, and severe in 14.1%. Duration since diagnosis was relatively evenly distributed: 32.2% had been diagnosed for 1–3 years, 33.2% for 4–6 years, and 34.6% for ≥7 years.

Table 2 presents the distribution of mothers' knowledge regarding ASD across key domains, including its definition, causes, symptoms, and management. Overall, the mothers demonstrated a moderate level of knowledge (mean ± SD: 2.12 ± 0.48), with 38.5% classified as having good knowledge, 35.1% fair, and 26.4% low. Understanding of autism's definition varied; while nearly half (47.8%) recognized it as a developmental disorder affecting sensory, cognitive, and social functions, only 19.0% correctly rejected the misconception that it is a form of intellectual disability. Knowledge of ASD etiology was limited, with only 17.1% identifying genetic factors and 15.1% recognizing chemical agents as possible causes, though 41.5% acknowledged prenatal complications as a potential factor. Symptom awareness was relatively better, particularly for delayed language development (62.4%), repetitive behaviors (58.5%), and lack of danger apprehension (56.6%), whereas hyposensitivity to pain remained poorly understood (18.5%). Regarding management strategies, the majority of the mothers were aware of non-pharmacological interventions such as speech sessions (81.0%) and behavioral therapy (77.1%), while fewer (44.4%) identified drug therapy as a valid approach, suggesting greater familiarity and confidence in therapeutic over medical interventions.

**Table 2:** Mothers’ knowledge about ASD.

Variable	Frequency (*n* = 205)	Percentage (100%)	Mean ± SD
What is autism?			
1. It is a term given to one of the degrees of intellectual disability.			
No	101	49.3	1.70 ± 0.77
Uncertain	65	31.7	
Yes	39	19.0	
2. It is a disorder of progressive development characterized by limitations or stops in sensory perception, language, ability to communicate, cognitive and social development.			
No	76	37.1	2.11 ± 0.91
Uncertain	31	15.1	
Yes	98	47.8	
3. It is a disorder of social development in a child.			
No	74	36.1	2.08 ± 0.89
Uncertain	41	20.0	
Yes	90	43.9	
What are the causes of the disease?			
1. Genetic			
No	92	44.9	1.72 ± 0.73
Uncertain	78	38.0	
Yes	35	17.1	
2. Method of treatment of parents (socialization)			
No	77	37.6	1.95 ± 0.83
Uncertain	62	30.2	
Yes	66	32.2	
3. Chemical agents			
No	95	46.3	1.69 ± 0.72
Uncertain	79	38.5	
Yes	31	15.1	
4. Unfavorable conditions for the mother during the first 6 months of pregnancy			
No	82	40	2.01 ± 0.90
Uncertain	38	18.5	
Yes	85	41.5	
What are the symptoms of autism?			
1. Language development is slow			
No	53	25.9	2.37 ± 0.86
Uncertain	24	11.7	
Yes	128	62.4	
2. Spend little time with family members			
No	43	21.0	2.29 ± 0.79
Uncertain	59	28.8	
Yes	103	50.2	
3. Lack of interest in making friends with others			
No	58	28.3	2.15 ± 0.83
Uncertain	59	28.3	
Yes	88	42.9	
4. Tension increases if someone touches them			
No	72	35.1	2.06 ± 0.87
Uncertain	49	23.9	
Yes	84	41.0	
5. Hyposensitivity to pain			
No	102	49.8	1.69 ± 0.76
Uncertain	65	31.7	
Yes	38	18.5	
6. Imitate the movements of others			
No	62	30.2	2.07 ± 0.82
Uncertain	67	32.7	
Yes	76	37.1	
7. Lack of imaginative play			
No	61	29.8	2.13 ± 0.84
Uncertain	57	27.8	
Yes	87	42.4	
8. The child appears not to hear			
No	48	23.4	2.32 ± 0.83
Uncertain	43	21.0	
Yes	114	55.6	
9. Refusing to embrace others			
No	84	41.0	2.09 ± 0.95
Uncertain	19	9.3	
Yes	102	49.7	
10. Refusing to look into the eyes of those speaking to him/her			
No	63	30.7	2.18 ± 0.87
Uncertain	43	21.0	
Yes	99	48.3	
11. Repeating other people's words			
No	76	37.1	2.06 ± 0.89
Uncertain	40	19.5	
Yes	89	43.4	
12. Resistance to change in the usual regime			
No	68	33.2	1.99 ± 0.80
Uncertain	72	35.1	
Yes	65	31.7	
13. Have repeating movements			
No	43	21.0	2.38 ± 0.81
Uncertain	42	20.5	
Yes	120	58.5	
14. Apprehension of danger			
No	34	16.6	2.40 ± 0.75
Uncertain	55	26.8	
Yes	116	56.6	
What are the management methods for a child with autism?			
1. Drug therapy			
No	46	22.4	2.22 ± 0.78
Uncertain	68	33.2	
Yes	91	44.4	
2. Behavioral therapy			
No	18	8.8	2.68 ± 0.62
Uncertain	29	14.1	
Yes	158	77.1	
3. Speech sessions			
No	34	16.6	2.64 ± 0.75
Uncertain	5.0	2.4	
Yes	166	81.0	
Overall assessment of knowledge			
Low	54	26.4	2.12 ± 0.48
Fair	72	35.1	
Good	79	38.5	

Table 3 illustrates mothers' home care practices across four key domains, namely, nutrition, personal hygiene, motor skills, and safety measures, with the overall findings indicating high levels of maternal involvement in supporting child development. The majority of mothers demonstrated high engagement in nutrition (74.6%, mean ± SD: 2.67 ± 0.59), motor skills (74.1%, mean ± SD: 2.68 ± 0.56), and personal hygiene (69.2%, mean ± SD: 2.59 ± 0.66), while safety practices showed comparatively lower adherence (47.3%, mean ± SD: 2.32 ± 0.72). Nutrition-related practices such as encouraging children to remain seated during meals (87.8%) and using cups or straws (83.9%) were commonly implemented, whereas interactive methods such as using dolls or discussing food through pictures were less frequently practiced. In personal hygiene, the majority of the mothers trained their children to wash their hands (72.7%) and manage postbath care (68.3%), though tooth brushing and nasal hygiene were slightly less emphasized. Motor skill development was strongly supported through activities such as balancing (72.7%) and writing tool use (71.7%), but fine motor tasks such as bead threading were less common (21.0%, mean ± SD: 1.73 ± 0.78). Safety practices were moderate overall; although some mothers consistently stored sharp tools safely (44.9%) and taught basic emergency responses (35.6%), fewer adhered to critical measures such as locking away hazardous materials (22.0%) or using safety gates (26.3%).

**Table 3:** Mothers’ home care practices for nutrition, personal hygiene, motor skills, and safety measures.

Variable	Frequency (*n* = 205)	Percentage (100%)	Mean ± SD
Mothers’ home care practices for nutrition			
1. I train the child to participate in bringing and placing food			
Never	11	5.4	2.59 ± 0.59
Sometimes	62	30.2	
Always	132	64.4	
2. I train the child to feed themselves			
Never	15	7.3	2.54 ± 0.63
Sometimes	65	31.7	
Always	125	61.0	
3. I train the child to use a cup to drink and to use a straw to drink liquids			
Never	14	6.8	2.77 ± 0.56
Sometimes	19	9.3	
Always	172	83.9	
4. I encourage the child to stick to firm sitting until the meal is finished			
Never	14	6.8	2.81 ± 0.54
Sometimes	11	5.4	
Always	180	87.8	
5. I arrange eating times with consideration of the child's needs and feelings of hunger			
Never	14	6.8	2.62 ± 0.61
Sometimes	49	23.9	
Always	142	69.3	
6. I discuss eating with the child through pictures.			
Never	48	23.4	1.98 ± 0.66
Sometimes	114	55.6	
Always	43	21.0	
7. I encourage the child to express themselves and their feelings about eating			
Never	51	24.9	2.20 ± 0.81
Sometimes	62	30.2	
Always	92	44.9	
8. I use dolls (the bride) and play to express their reactions to food and its types.			
Never	88	42.9	1.80 ± 0.78
Sometimes	70	34.1	
Always	47	22.9	
Overall assessment of mothers’ home care practices for nutrition			
Low (1–1.66)	14	6.8	2.67 ± 0.59
Moderate (1.67–2.33)	38	18.6	
High (>2.33)	153	74.6	
Mothers’ home care practices for personal hygiene			
1. I train the child to wash their hands before and after eating			
Never	8.0	3.9	2.69 ± 0.54
Sometimes	48	23.4	
Always	149	72.7	
2. I train the child to brush their teeth			
Never	24	11.7	2.41 ± 0.69
Sometimes	72	35.1	
Always	109	53.2	
3. I train the child to clean their nose			
Never	32	15.6	2.44 ± 0.74
Sometimes	51	24.9	
Always	122	59.5	
4. I train the child to clean and take care of themselves after the bath			
Never	28	13.7	2.55 ± 0.72
Sometimes	37	18.0	
Always	140	68.3	
Overall assessment of mothers’ home care practices for personal hygiene			
Low (1–1.66)	20	9.8	2.59 ± 0.66
Moderate (1.67–2.33)	43	21.0	
High (>2.33)	142	69.2	
Mothers’ home care practices for motor skills			
1. I train the child in some simple exercises, such as running and jumping			
Never	27	13.2	2.47 ± 0.71
Sometimes	55	26.8	
Always	123	60.0	
2. I train the child to hold a pencil and a crayon			
Never	15	7.3	2.64 ± 0.61
Sometimes	43	21.0	
Always	147	71.7	
3. I train the child to join the big beads			
Never	99	48.3	1.73 ± 0.78
Sometimes	63	30.7	
Always	43	21.0	
4. I train the child to walk in balance			
Never	16	7.8	2.65 ± 0.62
Sometimes	40	19.5	
Always	149	72.7	
5. I train the child to reduce their stereotypical repetitive movements			
Never	28	13.7	2.29 ± 0.69
Sometimes	90	43.9	
Always	87	42.4	
6. I encourage the child to participate in various motor activities			
Never	32	15.6	2.23 ± 0.70
Sometimes	94	45.9	
Always	79	38.5	
Overall assessment of mothers’ home care practices for motor skills			
Low (1–1.66)	11	5.4	2.68 ± 0.56
Moderate (1.67–2.33)	42	20.5	
High (>2.33)	152	74.1	
Mothers’ home care practices for safety measures			
1. I install high locks on all doors and windows that are difficult for the child to reach, especially those leading to the street or dangerous areas (such as the roof or a pool)			
Never	42	20.5	2.07 ± 0.69
Sometimes	107	52.2	
Always	56	27.3	
2. I use safety gates at the entrances to rooms containing dangerous materials or unsafe areas (such as stairs or the kitchen)			
Never	49	23.9	2.02 ± 0.71
Sometimes	102	49.8	
Always	54	26.3	
3. I keep all cleaning supplies, medicines, and chemicals in securely locked cabinets high up and out of children's reach			
Never	76	37.0	1.85 ± 0.75
Sometimes	84	41.0	
Always	45	22.0	
4. I arrange furniture so it does not obstruct the child's movement or cause them to trip. I secure heavy furniture (like cabinets and TV units) to the wall to prevent them from falling			
Never	53	25.9	2.01 ± 0.72
Sometimes	97	47.3	
Always	55	26.8	
5. I teach the child, as much as possible and according to their abilities, how to act in simple emergencies, such as how to ask for help			
Never	68	33.2	2.02 ± 0.83
Sometimes	64	31.2	
Always	73	35.6	
6. I store sharp tools (knives, scissors) in safe places and cover all electrical outlets with protective covers			
Never	47	22.9	2.22 ± 0.79
Sometimes	66	32.2	
Always	92	44.9	
Overall assessment of mothers’ home care practices for safety measures			
Low (1–1.66)	31	15.1	2.32 ± 0.72
Moderate (1.67–2.33)	77	37.6	
High (>2.33)	97	47.3	

Table 4 demonstrates that mothers' knowledge about autism ASD was significantly associated with several sociodemographic and clinical characteristics (*p* < 0.05). Higher knowledge levels were observed among mothers aged 29–38 years, those with higher education (particularly bachelor's and postgraduate degrees), urban residents, and those from smaller families or with sufficient income. Notably, consanguineous marriage and a positive family psychiatric history were also linked to greater knowledge. Although counterintuitive, the majority of the mothers who attended autism education sessions only had fair knowledge. Clinically, knowledge levels were higher among mothers of second-born children with autism, those caring for children with severe symptoms, and those whose child had been diagnosed 4–6 years prior. In contrast, no significant associations were found with the child's gender or age (*p* > 0.05).

**Table 4:** Association between the mothers’ knowledge about autism and the sociodemographic and clinical characteristics of the study participants (*n* = 205).

Variable	Overall knowledge category			*P*-value
	Low *N* = 54 (26.4%)	Fair *N* = 72 (35.1%)	Good *N* = 79 (38.5%)	
Mother's age (years)				
18–28	4.0 (16.7)	16 (66.6)	4.0 (16.7)	**0** **.** **004**
29–38	27 (27.0)	26 (26.0)	47 (47.0)	
≥39	23 (28.4)	30 (37.0)	28 (34.6)	
Education level				
Cannot read/write	0.0 (0.0)	3.0 (37.5)	5.0 (62.5)	**0** **.** **001**
Reads and writes	13 (65.0)	0.0 (0.0)	7.0 (35.0)	
Elementary	6.0 (50.0)	5.0 (41.7)	1.0 (8.3)	
Secondary	26 (44.8)	16 (27.6)	16 (27.6)	
Diploma	5.0 (11.6)	20 (46.5)	18 (41.9)	
Bachelor	4.0 (7.3)	25 (45.5)	26 (47.2)	
Postgraduate	0.0 (0.0)	3.0 (33.3)	6.0 (66.7)	
Place of residence				
Urban	51 (27.3)	58 (31.0)	78 (41.7)	**0** **.** **001**
Rural	3.0 (16.7)	14 (77.8)	1.0 (5.6)	
Family size				
3–5 members	21 (17.6)	41 (34.5)	57 (47.9)	**0** **.** **001**
6–9 members	21 (42.0)	10 (20.0)	19 (38.0)	
≥10 members	12 (33.3)	21 (58.3)	3.0 (38.4)	
Monthly income				
Not enough	15 (26.8)	36 (64.3)	5.0 (8.9)	**0** **.** **001**
Somewhat enough	26 (37.1)	5.0 (7.1)	39 (55.8)	
Enough	13 (16.5)	31 (39.2)	35 (44.3)	
Family psychiatric history				
Yes	13 (46.4)	3.0 (10.7)	12 (42.9)	**0** **.** **005**
No	41 (23.2)	69 (39.0)	67 (37.8)	
Consanguineous marriage				
Yes	6.0 (7.7)	25 (32.1)	47 (60.2)	**0** **.** **001**
No	48 (37.8)	47 (37.0)	32 (25.2)	
Attended autism education sessions				
Yes	0.0 (0.0)	26 (89.7)	3.0 (10.3)	**0** **.** **001**
No	54 (30.7)	46 (26.1)	76 (43.2)	
Child's gender				
Male	30 (22.6)	54 (40.6)	49 (36.8)	0.061
Female	24 (33.3)	18 (25.0)	30 (41.7)	
Child's age (years)				
2–7	30 (23.3)	44 (34.1)	55 (42.6)	0.249
8–13	21 (34.4)	20 (32.8)	20 (32.8)	
14–16	3.0 (20.0)	8.0 (53.3)	4.0 (26.7)	
Birth order of the child with autism				
Only child	0.0 (0.0)	4.0 (50.0)	4.0 (50.0)	**0** **.** **001**
First	33 (42.3)	33 (42.3)	12 (15.4)	
Second	3.0 (11.5)	3.0 (11.5)	20.0 (77.0)	
Third or more	18 (19.4)	32 (34.4)	43 (46.2)	
Severity of autism				
Mild	11 (21.2)	18 (34.6)	23 (44.2)	**0** **.** **002**
Moderate	30 (24.2)	53 (42.7)	41 (33.1)	
Severe	13 (44.8)	1.0 (3.4)	15 (51.8)	
Duration since diagnosis				
1–3 years	14 (21.2)	40 (60.6)	12 (18.2)	**0** **.** **001**
4–6 years	7.0 (10.3)	16 (23.5)	45 (66.2)	
≥7 years	33 (46.5)	16 (22.5)	22 (31.0)	

The bold values provided that the *P*-value was <0.05 and its statistically significant.

Table 5 presents the association between mothers' knowledge levels about ASD and their home care practices across the following four domains: nutrition, personal hygiene, motor skills, and safety measures. The findings reveal statistically significant associations (*p* < 0.001) in all domains. In terms of nutrition, all the mothers with low and moderate practice scores were classified within the fair or good knowledge categories, with 76.4% of those demonstrating moderate practices and 73.4% of those with high practices having good knowledge, respectively. Similarly, personal hygiene practices improved with increasing knowledge: 86.1% of mothers with moderate hygiene practices and 64.5% with high practices fell within the good knowledge group. A comparable pattern was observed in motor skill development, where 87.3% of mothers with high practice scores demonstrated good knowledge, and none of the mothers with low practice levels had good knowledge. For safety measures, a progressive increase in knowledge was associated with higher levels of practice; 69.6% of mothers with high safety practices exhibited good knowledge, while 53.7% of those with low safety practices only had fair knowledge, and 37.0% fell within the low knowledge category.

**Table 5:** Association between the mothers’ knowledge and home care practices for nutrition, personal hygiene, motor skills, and safety measures for children with autism.

Variable	Overall knowledge category *N* = 205 (100%)			*P*-value
	Low *N* = 54 (26.4%)	Fair *N* = 72 (35.1%)	Good *N* = 79 (38.5%)	
Overall assessment of mothers’ home care practices for nutrition				
Low (1–1.66)	14 (25.9)	0.0 (0.0)	40 (74.1)	**0** **.** **001**
Moderate (1.67–2.33)	0.0 (0.0)	17 (23.6)	55 (76.4)	
High (>2.33)	0.0 (0.0)	21 (26.6)	58 (73.4)	
Overall assessment of mothers’ home care practices for personal hygiene				
Low (1–1.66)	10 (18.5)	15 (27.8)	29 (53.7)	**0** **.** **001**
Moderate (1.67–2.33)	3.0 (4.2)	7.0 (9.7)	62 (86.1)	
High (>2.33)	7.0 (8.9)	21 (26.6)	51 (64.5)	
Overall assessment of mothers’ home care practices for motor skills				
Low (1–1.66)	7.0 (13.0)	18 (33.3)	29 (53.7)	**0** **.** **001**
Moderate (1.67–2.33)	4.0 (5.6)	14 (19.4)	54 (75.0)	
High (>2.33)	0.0 (0.0)	10 (12.7)	69 (87.3)	
Overall assessment of mothers’ home care practices for safety measures				
Low (1–1.66)	20 (37.0)	29 (53.7)	5.0 (9.3)	**0** **.** **001**
Moderate (1.67–2.33)	7.0 (9.7)	28 (38.9)	37 (51.4)	
High (>2.33)	4.0 (5.1)	20 (25.3)	55 (69.6)	

The bold values provided that the *P*-value was <0.05 and its statistically significant.

## Discussion

This study assessed maternal knowledge and home care practices for children with ASD in Diwaniyah, Iraq, revealing important insights within a context of limited autism-specific resources, socioeconomic challenges, and cultural influences. Maternal knowledge about ASD was moderate, with 38.5% demonstrating good knowledge and 26.9% showing low knowledge. These findings align with Wang et al. (7), who reported that caregivers often possess a basic understanding but lack comprehensive awareness of ASD's causes and management. Similarly, Othman et al. (8) observed increased parental knowledge after educational programs, though baseline knowledge varied. Dwairy (14) highlighted persistent misconceptions among Arab parents, particularly attributing ASD to supernatural causes; while less prominent in our cohort, cultural beliefs still shape understanding. The urban setting of Diwaniyah may explain the relatively better knowledge observed compared to rural areas.

Regarding home care practices, the mothers were highly engaged in nutrition (85.3%), personal hygiene (74.2%), and motor skill activities (67.7%), but engagement in safety practices was lower (40.5%). This pattern mirrors previous studies (15–17) that report the prioritization of nutrition and hygiene over safety. The low emphasis on safety is concerning, as children with ASD are particularly vulnerable to injury, and only 40% of mothers recognized impaired danger awareness as a symptom. This underscores the need for targeted home safety education.

Higher maternal knowledge was significantly associated with education level, urban residence, family size, and monthly income, consistent with prior research (18). Interestingly, consanguinity also correlated positively with maternal knowledge, possibly reflecting increased exposure to genetic counseling or heightened awareness in families with hereditary health issues, a relevant factor in Iraqi society. The mothers demonstrated strong awareness of observable symptoms such as delayed language and repetitive behaviors (over 60%), but knowledge regarding ASD etiology and long-term management was limited, indicating a symptom-focused understanding rather than comprehension of the condition's complexity. This aligns with findings from similar studies, where mothers also showed good recognition of core ASD symptoms (19–22).

Awareness of non-pharmacological interventions, including speech therapy and behavioral strategies, was relatively high (70%), likely influenced by local autism centers. However, knowledge of individualized therapy planning was limited, suggesting a need to expand education beyond basic therapy options. While engagement in motor skill development, nutrition, and hygiene was strong, home care practices related to safety were insufficient. Unsafe home environments, such as poorly stored hazardous materials, absence of safety gates, and inadequate emergency preparedness, pose significant risks for children with ASD, consistent with findings from other low-resource settings (23).

A positive correlation between maternal knowledge and home care practices was observed across all domains, highlighting the critical role of knowledge in shaping care. This supports international evidence that higher caregiver knowledge improves care quality and child outcomes (24, 25). Notably, attendance at autism educational sessions did not significantly enhance maternal knowledge, suggesting that current programs in Diwaniyah may be too generic, overly didactic, or not aligned with mothers' practical challenges. In contrast, structured, interactive, culturally sensitive programs have improved caregiver knowledge in other regions (26). Future interventions should incorporate visual aids, real-life scenarios, and community-specific concerns.

To improve ASD care in Iraq, policymakers should prioritize culturally sensitive education programs that enhance both knowledge and practical skills, correct misconceptions, and promote home safety. Efforts should extend diagnostic and support services beyond urban centers, train healthcare providers in rural areas, and integrate home safety support into national policies. Given the high prevalence of consanguinity, integrating genetic counseling into ASD care pathways is essential. Future research should adopt longitudinal designs to clarify causal relationships between maternal knowledge and home care practices, include fathers and other family members, and evaluate tailored educational interventions. Investigating barriers to home safety and expanding research on behavioral management, communication facilitation, and caregiver psychological support will provide a more comprehensive understanding of caregiving environments.

### Strengths and limitations

This study has several strengths. The use of a census sampling method included all eligible mothers registered at key autism centers in Diwaniyah, enhancing representativeness within this urban population. The interviewer-administered questionnaire, validated for cultural relevance, ensured comprehensive and reliable data collection across multiple caregiving domains. Additionally, a pilot study was conducted to refine the questionnaire, further improving data quality. Nevertheless, several limitations should be noted. The cross-sectional design limits causal inference between maternal knowledge and caregiving practices. The sample was restricted to mothers attending specialized autism centers, potentially excluding caregivers without access to such services and limiting generalizability to rural or underserved populations. The study did not assess the roles of fathers or other family members, which could provide a more holistic understanding of caregiving dynamics. Furthermore, some key caregiving aspects, such as communication facilitation and behavioral management strategies, were not explored. Additionally, the use of interviewer-administered questionnaires may have introduced social desirability bias, whereby participants provide responses perceived as favorable rather than fully accurate.

## Conclusion

This study demonstrates that mothers of children with ASD in Diwaniyah exhibit moderate knowledge and varying engagement in home care practices, with strengths in nutrition and hygiene but notable gaps in safety measures. Maternal education, socioeconomic factors, and consanguinity significantly influence knowledge levels. The strong association between knowledge and caregiving practices highlights the critical need for targeted, culturally tailored educational programs that not only improve understanding but also enhance practical caregiving skills, particularly in home safety. Policymakers and healthcare providers must prioritize expanding accessible autism services and developing comprehensive support frameworks to improve outcomes for children with ASD and their families in Iraq.

## Data Availability

The raw data supporting the conclusions of this article will be made available by the authors, without undue reservation.
